# The Origins of Salivary Vitamin A, Vitamin B_12_ and Vitamin D-Binding Proteins

**DOI:** 10.3390/nu12123838

**Published:** 2020-12-16

**Authors:** Matthew Blakeley, Agata Sobczyńska-Malefora, Guy Carpenter

**Affiliations:** 1 Salivary Research, Centre for Host-Microbiome Interactions, Faculty of Dental, Oral and Craniofacial Sciences (FoDOCS), King’s College London, London SE1 1UL, UK; matthew2@kth.se; 2 The Nutristasis Unit, Thomas’ Hospital, Viapath, St., London SE1 7EH, UK; agata.malefora@viapath.co.uk; 3 Faculty of Life Sciences and Medicine, King’s College London, London SE1 9NH, UK

**Keywords:** vitamins, saliva, haptocorrin, vitamin D-binding protein, retinol-binding protein

## Abstract

Vitamin A- (retinol), vitamin B_12_- (haptocorrin) and vitamin D-binding proteins are the major circulatory transporters of their respective ligands; they are also constituents of the salivary proteome, the origins of which, remain unclear. The aim of this study was to explore how these proteins enter saliva and their relationship (if any) with vitamin status. Firstly, the three vitamin-binding proteins were quantified in resting whole mouth saliva and chewing-stimulated saliva from healthy donors (*n* = 10) to determine if they enter the mouth by salivary secretion or from the circulation. Secondly paired whole mouth saliva and serum samples were analysed from healthy donors (*n* = 14) to determine the relationships between the vitamin-binding proteins and vitamin status. Salivary output of all three vitamin-binding proteins studied increased when secretion was stimulated, suggesting they are secreted by the salivary glands. Whilst retinol-binding protein and haptocorrin were secreted by all major salivary glands, vitamin D-binding protein was restricted to the mucus glands. Salivary vitamin-binding protein concentrations were not found to be indicative of systemic vitamin status.

## 1. Introduction

The proteome of whole mouth saliva (WMS) is vast, consisting of over 2000 species, and sharing approximately 27% homology to that of plasma [1,2]. Proteins enter saliva via a number of routes, with the majority being expressed by salivary gland acinar compartments and secreted by the exocytotic pathway [3]. A number of proteins are translocated into WMS either actively by the salivary gland parenchyma, or passively from the gingival margin, where a serum exudate pools, termed gingival crevicular fluid (GCF) [4]. WMS is the collective product of the three bilaterally paired major salivary glands (parotid, sublingual, and submandibular); collectively these six glands are responsible for 90% of WMS volume which equates to 2 L over 24 h. The remaining volume is provided by the numerous minor glands distributed throughout the oral mucosa, as well as contributions from the GCF, oral microbiome, and the oral epithelium [5]. Many salivary constituents are discretely expressed by the various glands, and the regulation of those glands is controlled by the extrinsic stimuli and circadian rhythm [3,6]. This complex regulation in WMS flow and composition yields a dynamic biofluid which, in health, supports the diverse functions of the oral cavity [7].

A number of vitamin-binding proteins (VBP) are constituents of the salivary proteome. This study focuses on retinol-binding protein (RBP), haptocorrin (Hc), and vitamin D-binding protein (DBP), which are additionally present in the proteome of plasma [1]. Determining the origin of these proteins in saliva would allow their correct quantification and thus how they may relate to circulatory vitamin levels and their potential for indicating vitamin status. Although the function of these three proteins in the circulation has been well studied, their roles, particularly of RBP and DBP have been under explored in the context of the oral cavity [8,9,10,11].

In the circulation, vitamin A (retinol), and the provitamin A retinoids are highly hydrophobic and therefore have low solubility in blood but this is improved by binding RBP. The retinol–RBP complex further recruits another binding protein termed transthyretin; the large collective molecular weight prevents its removal from the circulation during glomerular filtration, thus enhancing the circulatory half-life of retinol [12]. In addition to its role in retinoid transport, it is also suggested that RBP is an adipokine, and an indicator of insulin resistance in type 2 diabetes [9,13]. Clinical assessment of an individual’s vitamin A status commonly involves measuring total retinol in the circulation which correlates well with RBP, however the relationship between vitamin A status and RBP concentration in other biofluids is unclear [14].

Hc, or R-binder, is a highly glycosylated protein with two domains, which binds vitamin B_12_ and its analogues, with low specificity [15]. In the circulation, 80–90% of vitamin B_12_ is bound to Hc, which circulates with almost fully saturated B_12_ binding sites. However, this fraction is not accessible to cells and a receptor for the complex is unknown [16,17,18]. The remaining 10–20% of B_12_ is bound to transcobalamin (TCN), and this is the biologically active fraction of B_12_ [18]. Due to being the major circulatory carrier of B_12_, and the high degree of saturation, Hc concentration correlates well with the commonly used marker of B_12_ status, total vitamin B_12_ [19,20,21]. Hc has been more widely detected in the proteomes of other biofluids including gastric fluid, lacrimal and nasal secretions, as well as breast milk and seminal plasma [22,23,24,25]. In the upper digestive tract, Hc binds dietary vitamin B_12_ and protects it against hydrolysis in the acidic milieu of the stomach [26]. Its low specificity between vitamin B_12_ analogues, and ubiquitous secretion onto mucosal surfaces, suggests it functions as an innate antimicrobial, possibly by sequestering analogues essential for many bacterial species; data to support this hypothesis however, are limited.

DBP, also referred to as Gc-globulin, is the major carrier of vitamin D metabolites in the circulation, binding ~75% [27,28]. Vitamin D denotes a group of hydrophobic sercosteroids and therefore their solubility in plasma is enhanced by binding DBP. DBP circulates in apparent excess with 97–98% of vitamin-binding sites being unoccupied at a healthy vitamin D level, possibly due to the multifunctionality of the protein [29,30]. Beyond vitamin D binding, DBP also sequesters free actin monomers, is a potent activator of macrophages, and enhances complement mediated neutrophil chemotaxis [8,31]. DBP is also detectable in plasma, GCF, and saliva [32]. It is widely agreed that 25-OH-D is a robust and commonly employed biomarker for vitamin D status, and previous studies have indicated significant positive correlations to plasma concentrations of DBP [33,34].

The aim of this study was to understand if these VBP are produced by salivary glands or are merely exudated from serum. Secondly, we address their potential use in predicting vitamin status.

## 2. Materials and Method

In order to address the aims of this study, cross sectional analyses were undertaken.

### 2.1. Sample Collection

Self-declared healthy staff and students were recruited internally at King’s College London to participate in this study. Volunteers were selected for participation if they were in good general health with no history of gastro-intestinal disorders, and not currently taking medication or vitamin supplements. All samples were collected in January 2018.

#### 2.1.1. Salivary Vitamin-Binding Proteins at Rest and Stimulated Flow

Five female and five male, apparently healthy, participants provided resting WMS samples by expectorating into a pre-weighed sterile tube for 10 min. Tubes were again re-weighed in order to calculate the flow rate (weight/time). One gram of saliva was assumed to equate to 1 mL throughout. Saliva flow was stimulated by chewing on flavourless paraffin gum, and again expectorating into a pre-weighed sterile tube for 10 min. The tubes were again re-weighed to calculate the flow rate. Debris was precipitated from all saliva samples by centrifugation at 13,000× *g* for 10 min, clarified saliva was aliquoted and stored at −80 °C until testing.

#### 2.1.2. Glandular Contributions to the Vitamin-Binding Protein Output of Whole Mouth Saliva

One female and one male participant were asked to provide resting and chew stimulated WMS samples as described above. Additionally, parotid gland secretions were collected by attaching Lashley cups (by suction) to the papillae of the Stensen’s ducts located on the buccal mucosa adjacent to the second molar on each side of the upper arch. Once Lashley cups were fitted, participants rinsed their mouths by swilling 10 mL of water before sample collection. For a duration of 10 min resting parotid saliva was collected via the Lashley cup apparatus, and WMS excluding the parotid secretion (we now term WMS xP) by expectoration into a pre-weighed sterile tube. This collection process was repeated, this time with chew stimulated flow with the use of flavourless paraffin gum. As described previously, flow rates were calculated, debris was precipitated, and sample aliquots stored at −80 °C until testing.

#### 2.1.3. Comparing Vitamin-Binding Proteins Concentration with Systemic Vitamin Status

Six and eight, self-reported, healthy female and male participants, respectively, were asked to refrain from consuming food or drink (excluding water) for eight hours prior to sample collection. Resting WMS samples were provided and processed as described above. Venous blood (17 mL) was collected into serum separator tubes (Becton, Dickinson and Company, Franklin Lakes, NJ, USA) with butterfly needle safety collection kits. As per manufacturer instructions, samples were centrifuged at 2000× *g* for 10 min, and the serum was then aliquoted and stored at −80 °C until testing.

### 2.2. Biochemical Analysis

#### 2.2.1. Quantification of Total Protein Content by Bicinchoninic Acid Assay

Total protein concentration of samples was determined by bicinchoninic acid assay (BCA) (Thermo Fisher Scientific, Inchinnan, UK) following manufacturer’s instructions. In brief, albumin standard was titrated to yield a 2–0 mg/mL range. Standards and samples were added to a 96-well plate in duplicate with the suggested concentration of working reagent, the plate was incubated at 37 °C for 30 min before measuring absorbance at 540 nm using iEMS 96-well microplate reader (Thermo Fisher Scientific, Inchinnan, UK). Output (mg/min) of total salivary protein was calculated (concentration (mg/mL) × salivary flow rate (mL/min)).

#### 2.2.2. Quantification of Vitamin-Binding Proteins by Enzyme-Linked Immunosorbent Assay

Concentrations of the three VBP were assayed in saliva and serum samples by commercially available enzyme-linked immunosorbent assays (ELISA) following the manufacturer’s guidelines and suggested sample dilutions. Assays for RBP (Thermo Fisher Scientific, Inchinnan, UK), Hc (Cusabio biotech Co Ltd., Houston, TX, USA), and DBP (R&D Systems Europe, Ltd., Abingdon, UK) were measured at 450 nm on an iEMS 96- well microplate reader (Thermo Fisher Scientific, Inchinnan, UK). Salivary outputs of the three VBP were calculated (concentration × salivary flow rate).

#### 2.2.3. Quantification of Retinol by High-Performance Liquid Chromatography

Retinol was quantified in serum samples as a marker of systemic vitamin A status. Quantification was undertaken by high performance liquid chromatography (HPLC) using the Waters Alliance 2695 HPLC system (Waters, Herts, UK) coupled with UV detector at 975 nm (Jasco, great Dunmow, UK). A BetaSil C18 reverse phase HPLC column (3 μm 100 × 4.6 mm) (Thermo Fisher Scientific, Inchinnan, UK) was used.

#### 2.2.4. Quantification of Total Vitamin B_12_ & 25-OH-D by Chemiluminescent Microparticle Immunoassay

Total vitamin B_12_, and 25-hydroxyvitamin D were quantified in serum samples as markers of systemic vitamin B_12_ and vitamin D status, respectively. Quantification was undertaken by chemiluminescent microparticle immunoassay (CMIA) on an ARCHITECT iSystem (Abbott Diagnostics, Maidenhead, UK). After pre-treatments steps which included the release of vitamins from their binding proteins, vitamins were bound to intrinsic factor (vitamin B_12_ assay) or anti-vitamin D (vitamin D assay) coated microparticles. Incubation and wash steps followed, before the addition of a conjugate containing acridinium tracer. After further incubation and washing, hydrogen peroxide was added to each sample cup inducing a chemiluminescent reaction which is measured in relative light units (RLUs). RLUs have an inverse relationship to the amount of analyte tested and the RLUs detected by the ARCHITECT iSystem optics.

### 2.3. Statistical Analysis

Statistical analysis was undertaken using Prism 7 for Mac OS X Version 7.0a 2 April 2016 (GraphPad Software, San Diego, CA, USA). Normality of data was tested with Q-Q plots and the Shapiro–Wilk test. Statistical difference between groups was determined by a paired *t*-test. Correlations between parameters were assessed by simple linear regression.

### 2.4. Ethical Approval

Ethical approval for this study was obtained from the Biomedical Sciences, Medicine, Dentistry and Natural and Mathematical Sciences subcommittee of the King’s College London Research Ethics Committee (Reference: BDM/14/15-61) and the study protocol was designed following the King’s College London Guidelines on Good Practice in Academic Research. In accordance with the Declaration of Helsinki (2013), participants provided informed consent, and all samples were handled in accordance with the Human Tissue Act (2004). 

## 3. Results

To examine the source of the VBP in saliva, concentrations were measured in resting and chewing-stimulated saliva (Table 1). If these proteins were leaking into WMS from the serum compartment via the gingival crevicular fluid, it would not be expected for their output to increase with increasing salivary flow.

In the first group of healthy subjects presented in Table 1 (five males; five females), the mean resting salivary flow rate was 0.7 mL/min, which significantly (*p* < 0.01) increased to 1.95 mL/min when stimulated with chewing flavourless paraffin gum. Inversely, upregulation of salivary flow demonstrated significant decreases in total protein concentration (*p* < 0.05); however, when taking into account the increased flow rate, total protein output increased significantly (*p* < 0.01).

**Figure 1:**
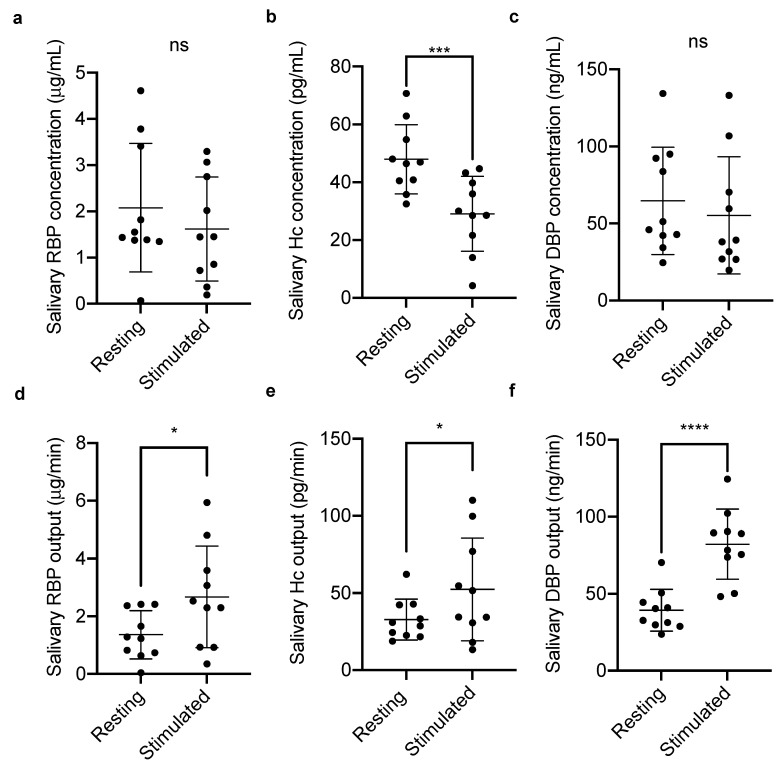
Whole mouth salivary (**a**,**d**) retinol-binding protein (RBP); (**b**,**e**) haptocorrin (Hc); and (**c**,**f**) vitamin D-binding protein (DBP) concentrations and outputs, at rest, and during chewing-stimulated flow. Individual measurements (*n* = 10) and corresponding means ± SD are displayed. Groups were compared by paired *t*-test (* *p* < 0.05, *** *p* < 0.001, **** *p* < 0.0001, ns = not significant). Correlation coefficients (**a**) *r* = 0.70, (**b**) *r* = 0.57, (**c**) *r* = 0.62, (**d**) *r* = 0.31, (**e**) *r* = 0.83, (**f**) *r* = 0.54.

RBP, Hc, and DBP concentrations and outputs were then compared in resting and chew-stimulated whole mouth saliva (Figure 1).

Of the three VBP measured, only the concentration of Hc significantly decreased when the salivary flow rate was upregulated by chewing; no significant differences were observed for DBP or RBP concentrations. The outputs of RBP, Hc, and DBP increased significantly between resting and stimulated flow with *p* values of <0.05, <0.05, and <0.0001, respectively.

**Figure 2:**
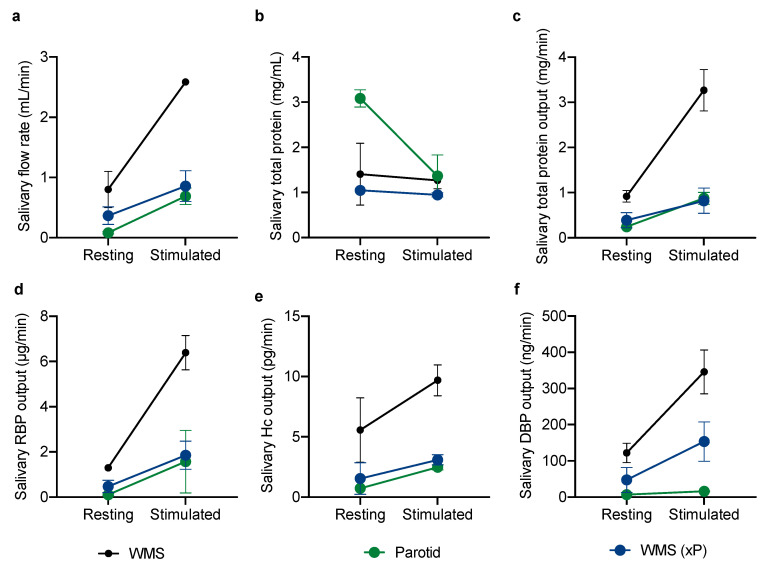
(**a**) The difference in whole mouth saliva (WMS); parotid; and whole mouth saliva excluding parotid (WMS (xP))-flow rates at rest and when stimulated by chewing. (**b**) The total protein concentration of the same samples. (**c**) Total protein as an output. The same samples were then assayed for (**d**) RBP; (**e**) Hc; and (**f**) DBP, which are displayed here as outputs. Points indicate the means of the two participants, with error bars indicating range.

To further understand the contribution of the different major salivary glands to the WMS VBP concentration, parotid saliva (P) was collected separately from submandibular and sublingual secretions. Whole mouth saliva samples were then compared to P and WMS without parotid saliva (WMS xP) at rest and when stimulated by chewing (Figure 2).

When compared to resting saliva flow rate, chewing upregulated flow rate in all study participants. In agreement with the first part of the study, the total protein concentration went down with increased flow rate, but the total protein output increased for all glands. Likewise, all VBP increased in output with chewing stimulation with the exception of parotid contribution to DBP which did not change from the resting output.

To further understand the origin of VBP, the salivary concentration and output for each salivary VBP was compared to its serum concentration, and vitamin status in a different group of 14 healthy participants (Table 2).

Serum concentrations of retinol, total vitamin B_12_, and 25-OH-D were all normally distributed when assessed by Q-Q plots, and the Shapiro–Wilk test. Retinol results ranged between 1.44 and 2.69 μmol/L, all patients were in the sufficient range of 1.4–3.84 μmol/L. Total vitamin B_12_ results ranged from 116 to 219 pmol/L, with three participants having results indicative of deficiency, <138 pmol/L [35]. 25-OH-D results ranged from 7 to 72 nmol/L; four participants had sufficient concentrations > 50 nmol/L, four participants had insufficient concentrations between 30 and 50 nmol/L, and five participants had deficient concentrations < 30 nmol/L. 

**Figure 3:**
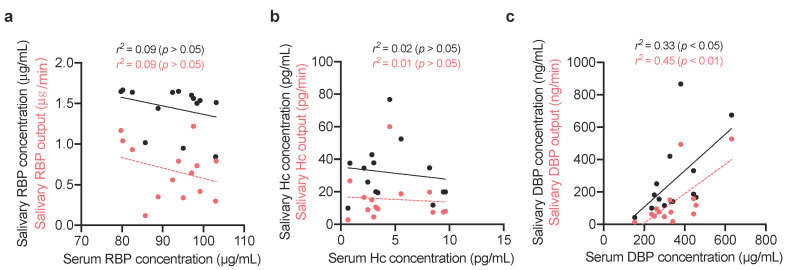
Linear regression between vitamin-binding proteins in serum and saliva. Individual measurements (*n* = 14) and the line of best fit are plotted. (**a**) Retinol-binding protein (RBP) (**b**) haptocorrin (Hc) (**c**) vitamin D-binding protein (DBP) concentrations in serum and saliva, and salivary outputs.

Of the three VBD assessed, only DBP correlated significantly between saliva and serum (Figure 3). Serum DBP correlated more strongly with salivary DBP output (*r*^2^ = 0.45, *p* < 0.01) than salivary concentration (*r*^2^ = 0.33, *p* < 0.05). Concentrations of the other salivary VBP did not show significant linear relationships with serum markers of vitamin status.

**Figure 4:**
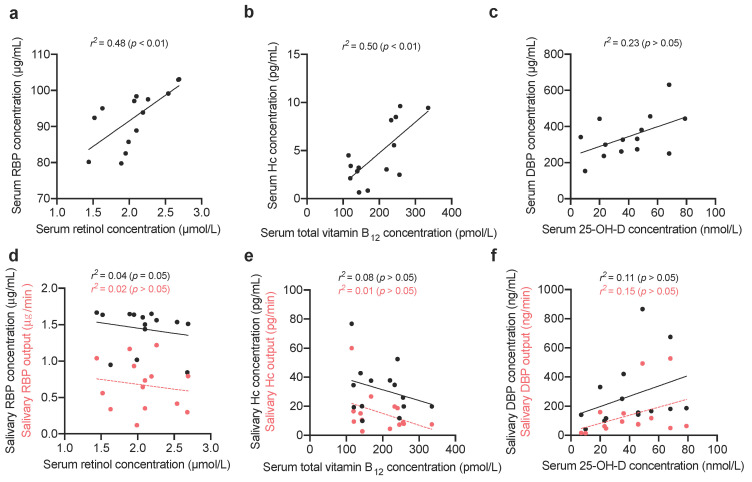
Linear regression between vitamin status and vitamin-binding proteins. Individual measurements (*n* = 14), and the line of best fit are plotted. (**a**) Serum retinol and serum retinol-binding protein (RBP) concentrations. (**b**) Serum total vitamin B_12_, and serum haptocorrin (Hc) concentrations. (**c**) Serum 25-OH-D, and serum vitamin D-binding protein (DBP) concentrations. (**d**) Serum retinol and salivary RBP concentration, and output. (**e**) Serum total vitamin B12, and salivary Hc concentration, and output. (**f**) Serum 25-OH-D and salivary DBP concentration, and output.

The salivary concentrations and outputs of the VBP were then compared to serum concentrations of their vitamins (Figure 4).

Serum RBP, and Hc significantly correlated with retinol (*r*^2^ = 0.48, *p* < 0.01) and total vitamin B_12_ (*r*^2^ = 0.50, *p* < 0.01), respectively, which complements previous studies [19,36]. Correlations have been shown previously between serum DBP and 25-OH-D; however, the relationship presented here is not significant [37]. No significant linear relationships were found between serum markers of vitamin status and salivary VBP concentrations or outputs.

## 4. Discussion

This study indicates that RBP, Hc, and DBP are secreted components of the salivary proteome, and their outputs are upregulated with chewing stimulated salivation. Chewing is known to increase salivary flow and protein secretion, thus the data presented here suggested these three proteins are locally synthesised and not just a serum contaminant; however, this should be confirmed with gene expression analysis of salivary gland tissue [38,39]. The majority of salivary proteins are known to be synthesised by the acinar compartment of the salivary glands, with a small contribution being made by the ductal compartment which also contains secretory granules [40]. This complements the decrease in salivary Hc concentration when salivary secretion is upregulated; additionally, immunohistochemical analysis of salivary gland tissue has previously shown Hc is localised to the ductal compartment [41]. Analysis of WMS, parotid and WMS xP in this study suggests that all major glands contribute to RBP and Hc output, however DBP is largely contributed by mucous glands. Inter-glandular variation in protein secretion has been noted before [42]. Amylase, for example, is the single most abundant protein in parotid secretion but forms less than 1% in submandibular saliva [43].

To the best of our knowledge, this is the first study to investigate the relationship between VBP in saliva and serum and vitamin status. We compared both salivary concentration and output to account for the large flow differences that occur between subjects and correlated them to serum markers of vitamin status. There appears to be no correlation between the salivary VBP concentration and vitamin status, which is in contrast to serum VBP, which has been shown previously to correlate to vitamin status [14,19,34]. However, salivary and serum DBP concentrations correlated significantly. Data in this study indicate that DBP is actively secreted by the salivary glands, and therefore the correlation between systemic and salivary DBP concentrations suggests that expression of the protein in the two biofluids is commonly regulated. Conversely, the lack of correlation between salivary and serum concentrations of RBP and Hc suggests salivary expression of these proteins is independent of their systemic levels.

This study raises questions regarding the function of VBP in saliva considering the high concentrations of the three proteins, and the lack of correlation between salivary and serum concentrations. Of the three proteins studied here, the function of Hc within the context of saliva has been the most researched. Assimilation of dietary vitamin B_12_ is a highly tuned process to scavenge a relatively scarce nutrient [17]. In humans, three cobalamin-binding proteins are responsible for vitamin B_12_ assimilation: Hc, intrinsic factor (IF), and TCN [17]. Salivary Hc is the first of these proteins to bind vitamin B_12_ upon its release from the food matrix, therefore offering protection from hydrolysis in the stomach. The vitamin is then liberated from Hc before binding IF which facilitates absorption in the ileum [26]. Following absorption, B_12_ is released into the blood; the majority of newly released B_12_ binds TCN (holoTC), which has a relatively short half-life (hours). TCN binds B_12_ with great specificity, only accepting vitamin B_12_ [15]. Hc is also present in serum and binds both vitamin B_12_ and inactive analogues; it has a significantly longer half-life (days). There is no evidence that Hc has any role in the cellular uptake of vitamin B_12_, however its role is still poorly understood [18]. One suggestion for its function is the transportation of potentially harmful vitamin B_12_ analogues to the liver for secretion into the bile, thus ensuring that only holoTC is available for cellular uptake, which is facilitated by binding the CD320 receptor [44,45]. 

The role of salivary RBP and DBP in vitamin assimilation has been underexplored in the literature. Unlike vitamin B_12_, vitamins A and D are both lipid soluble, and therefore their fate during digestion is different. Both these vitamins are found within the lipid fraction of the digest, and are incorporated into mixed micelles, before being actively transported into enterocytes by cholesterol receptors [46,47]. It is possible that salivary RBP and DBP offer an alternative pathway for enteric absorption.

Resting salivary flow occurs as a reflex and provides the oral cavity with saliva to maintain homeostasis [38]. Data presented here show that salivary Hc concentration is high at resting flow. It has been suggested that mucosal Hc may also have an antimicrobial function, due to the microbial requirement of vitamin B_12_ [48]. It can be hypothesised that Hc may function in a similar fashion to lactoferrin, which limits microbial growth by sequestering iron [49]. Studies on the gut microbiome indicate as much as 80% of species require B_12_ analogues but just 25% of those species have the complete biosynthesis machinery [50]. Consequently, it is becoming increasingly clear that vitamin B_12_ and its analogues are important modulators of biofilm structures on mucosal surfaces through microbial cross feeding and interdependence [50,51]. In addition to these studies, the extended human microbiome project published in 2017, highlighted an enrichment of vitamin B_12_ biosynthesis genes in the oral cavity demonstrating the significance of vitamin B_12_ to microbial metabolism [51]. Hc may therefore be an important factor of the innate immune system in sequestering B_12_ analogues and therefore reducing bioavailability to the oral microbiota, limiting growth. A number of the protein’s features make it suitable for this purpose. Firstly, of the three B_12_ binding proteins, Hc has the lowest specificity, thus binding structurally diverse analogues which can be utilised by bacteria. Secondly, Hc is widely detectable in a number of exocrine secretions including, saliva, breast milk, tears and seminal plasma, a number of which are secreted onto mucosal surfaces which have their associated microbiotas [22,23,25,52].

There are limited data to suggest the microbiota rely directly on exogenous vitamins A and D, however, some studies have shown that sufficient status of the host leads to healthier microbiota, likely due to indirect means such as immune system function [53,54]. It has been reported that DBP has important functions beyond vitamin transport. Studies have indicated that DBP has a synergistic effect with components of the complement pathway (C5a), therefore enhancing infiltration of neutrophils and monocytes [55,56]. Other studies have shown that sequential deglycosylation of DBP and β-galactosidase and sialidase secreted by immune cells produces a potent macrophage activating factor, upregulated phagocytic activity [57]. The high concentration of DBP in saliva may facilitate immunological functions and help maintain oral homeostasis.

There are a number of limitations to this study, primarily the limited sample size used. The data presented here offer a cursory look at the origin of VPS in saliva, and their relationship to vitamin status but larger studies are required. In addition, it was not possible to detect vitamins in saliva although attempts were made.

## 5. Conclusions

Retinol-binding protein, haptocorrin, and vitamin D-binding protein, are three constituents of the whole mouth saliva proteome. Evidence presented here suggests that retinol-binding protein and haptocorrin are secreted by all major salivary gland types; however, vitamin D-binding protein is restricted to the mucous glands. Salivary vitamin-binding protein concentrations are not indicative of whole-body vitamin status as assessed by standard techniques. 

## Figures and Tables

**Table 1:** Salivary flow rate and total protein concentration and output at rest and during chew stimulation.

*n* = 10	Resting		Stimulated			
	Mean	SD	Mean	SD	*p*	*r*
Flow rate (mL/min)	0.70	0.28	1.95	0.92	<0.01	0.44
Total protein concentration (mg/mL)	1.66	0.50	1.29	0.36	<0.05	0.66
Total protein output (mg/min)	1.85	0.26	2.35	0.98	<0.01	0.45

*p* values from a paired *t*-test. *r* correlation coefficient.

**Table 2:** The concentration of serum markers of vitamin status.

*n* = 14	Mean	SD
Serum retinol (μmol/L)	2.08	0.39
Serum total vitamin B_12_ (pmol/L)	196	92
Serum 25-OH-D (nmol/L)	40	22
